# Use of a Mobile Peer Support App Among Young People With Nonsuicidal Self-injury: Small-scale Randomized Controlled Trial

**DOI:** 10.2196/26526

**Published:** 2022-01-10

**Authors:** Kaylee Payne Kruzan, Janis Whitlock, Natalya N Bazarova, Aparajita Bhandari, Julia Chapman

**Affiliations:** 1 Center for Behavioral Intervention Technologies Northwestern University Chicago, IL United States; 2 Bronfenbrenner Center for Translational Research Cornell University Ithaca, NY United States; 3 Department of Communication Cornell University Ithaca, NY United States

**Keywords:** nonsuicidal self-injury, randomized controlled trial, mobile app, peer support, urges, digital intervention

## Abstract

**Background:**

Nonsuicidal self-injury (NSSI) is a widespread behavior among adolescents and young adults. Although many individuals who self-injure do not seek treatment, there is evidence for web-based help-seeking through web-based communities and mobile peer support networks. However, few studies have rigorously tested the efficacy of such platforms on outcomes relevant for NSSI recovery.

**Objective:**

The aim of this small-scale preregistered randomized controlled trial is to provide preliminary insight into the shorter- and longer-term efficacy of the use of a peer support app, TalkLife, in reducing NSSI frequency and urges and increasing readiness to change. In addition, we explore contact with informal support, interest in therapy, and attitudes toward professional help–seeking.

**Methods:**

Individuals aged 16-25 years with current (within 3 months) and chronic (>6 episodes in the past year) NSSI history were eligible to participate in this study. After baseline assessments, the intervention group was instructed to use the app actively (eg, post or comment at least three times per week) and the control group received weekly psychoeducational materials through email, for 8 weeks. Follow-up was assessed at 1 month and 2 months. Linear mixed modeling was used to evaluate condition and time point effects for the primary outcomes of NSSI frequency and urges, readiness to change, contact with informal support, interest in therapy, and attitudes toward professional help–seeking.

**Results:**

A total of 131 participants were included in the analysis. We evidenced a significant effect of condition on NSSI frequency such that the participants using the peer support app self-injured less over the course of the study (mean 1.30, SE 0.18) than those in the control condition (mean 1.62, SE 0.18; P=.02; *η*^2^=0.02). We also evidenced a significant condition effect of readiness to change such that the treatment participants reported greater confidence in their ability to change their NSSI behavior (mean 6.28, SE 0.41) than the control participants (mean 5.67, SE 0.41; P=.04; *η*^2^=0.02). No significant differences were observed for contact with informal support, interest in therapy, or attitudes toward professional help–seeking.

**Conclusions:**

Use of the peer support app was related to reduced NSSI frequency and greater confidence in one’s ability to change NSSI behavior over the course of the study period, but no effects on NSSI urges, contact with informal support, interest in therapy, or attitudes toward professional help–seeking were observed. The findings provide preliminary support for considering the use of mobile peer support apps as a supplement to NSSI intervention and point to the need for larger-scale trials.

**Trial Registration:**

Open Science Foundation; https://osf.io/3uay9

## Introduction

### Overview

Nonsuicidal self-injury (NSSI)—“the deliberate damage of body tissue without suicidal intent” [1]—is estimated to affect 17%-18% of young people [2]. Although NSSI often signals significant underlying distress and is a risk factor for future suicidal ideation and attempts [3-5], it can be a relatively invisible condition. Approximately half of the young people who self-injure do not disclose their NSSI thoughts or behaviors to anyone, [6] and those who choose to disclose them often do so only to close peers or parents [7-9]. Research has also shown similar rates of disclosure among those engaged in therapy, with approximately half discussing NSSI with their providers [9]. Thus, many young people who engage in NSSI do not access, or benefit from, resources to support NSSI behavior change. However, there is a rich exchange of information and social support related to NSSI on the web [10-12], and research suggests that many individuals who self-injure are receptive to web-based and mobile interventions [13-15]. Mobile apps that include elements of social support may thus be a promising direction for NSSI intervention.

### Background

#### Web-Based Communities and NSSI

Web-based peer-to-peer communication regarding NSSI on social media websites and social support forums is highly prevalent. A robust body of work demonstrates the exchange of informational support, such as strategies to cope with symptoms, resources, advice on help-seeking, as well as emotional support, such as validation of shared struggles and empathetic responses on web-based NSSI forums [16,17]. Commonly, benefits to participation in web-based communities for NSSI include an increased sense of belonging and social connection in a space that is free of the stigmas that may be encountered and inhibit help-seeking in day-to-day life. Indeed, a primary benefit of web-based spaces where individuals discuss NSSI seems to be the exchange of experiential knowledge and the sense of community this exchange affords [18]. Relationships in these web-based spaces are often cited as being destigmatizing and have been associated with decreased feelings of isolation, greater sense of purpose, and feelings of acceptance and belonging [19]. As web-based communication regarding NSSI has been linked to components deemed important for NSSI recovery (eg, sense of belonging and social connection) [20], it is possible that participation may increase one’s readiness to change NSSI behavior and contribute to the recovery process.

At the same time, there are noted risks to participation on such web-based forums for individuals with a history of NSSI behavior. The types of information exchanged on the web are not always reliable or congruent with existing therapeutic or clinical recommendations [21]. Moreover, participation in web-based communities can lead to the normalization of NSSI behavior [22-24] or expose individuals to triggering graphic or emotional images or text [17,25,26].

Although qualitative work has set a foundation for understanding the likely effects of the exchange of peer support on NSSI behavior, few studies have examined this relationship in a controlled trial. Some social media research suggests that high levels of use of [27], and exposure to, NSSI content may be related to increased odds of NSSI behavior over time [28]. Preliminary evidence from survey research suggests that engagement in peer support communities may be linked to reductions in NSSI behavior [29], and positive interpersonal relationships are protective against both NSSI behavior and worsening symptoms [30-32], as well as a facilitator of NSSI recovery [20]. Given the high rates of web-based activity related to NSSI, particularly among young people who are otherwise unlikely to engage in treatment, there is a need for further empirical tests of the relationship between web-based peer support and NSSI outcomes.

#### Utility of Mobile Apps for NSSI

A growing body of research provides support for the efficacy of web-based and mobile apps in reducing various mental health symptoms [33-36]. Although the structure and goals of these digital interventions vary (eg, symptom tracking, therapy, coaching, assessment, and peer support) [37], support for the benefits of mobile apps over no-treatment controls is consistent [38], making them a useful alternative for individuals who face barriers to traditional in-person treatment, such as stigma, cost, and accessibility [39,40].

For individuals who have unaddressed NSSI behaviors, web-based and mobile apps may provide some relief and serve as gateways for additional help. Young people report interest in, and acceptability of, digital interventions for NSSI [41-44]. However, as in the case of peer support forums, there are few efficacy trials of digital interventions for NSSI. A recent review of the effectiveness of web-based and mobile apps for self-injurious thoughts and behaviors broadly conceived (with and without suicidal intent) showed limited evidence for their efficacy in reducing NSSI [13,45]. However, several apps have been associated with encouraging preliminary findings.

Franklin et al [46] conducted 3 randomized controlled trials on a mobile intervention that used an aversive conditioning approach with images related to NSSI. The intervention was associated with fewer self-cutting episodes over the treatment period, but there were no effects on other outcomes (eg, suicidal ideation or dysregulated emotion) and treatment effects were not retained at 1-month follow-up. The mobile app Blue Ice was designed as an adjunct to face-to-face therapy, and its primary function is to link users to coping strategies rooted in cognitive behavioral therapy (CBT) and dialectical behavior therapy, including a mood diary, mood-lifting activities, and safety checks to prevent self-harm [47]. A preliminary trial showed postuse improvement in depression and anxiety symptoms, and 73% of the users reported having stopped or decreased self-injury over the course of the study. Similarly, pilot trials of another adjunctive skills-based app (DBT Coach) demonstrated reductions in urges to self-harm, NSSI frequency, and subjective distress [48], as well as increases in self-efficacy and emotion regulation among individuals with borderline personality disorder [49].

Although extant research suggests the feasibility and acceptability of mobile apps for treating NSSI behaviors, few publicly available apps have been evaluated through efficacy trials. Most of the apps reviewed have focused on internet-based CBT, psychoeducation, or elements of *third wave* CBT, including mindfulness and acceptance [45]. Despite the prevalence of web-based peer-to-peer communication regarding NSSI, none of these studies of apps included or evaluated a peer support component. In sum, there have been no trials, to our knowledge, that examine the relationship between web-based peer support exchange and NSSI outcomes.

### Objectives

This study explores the efficacy of a mobile peer support app, TalkLife, in improving NSSI outcomes and informal support and formal help-seeking outcomes. This app is free and publicly available and designed to provide young people with immediate and informal mental health support. Preliminary research on this app shows that many young people use it to discuss NSSI and related mental health conditions [11,50]. A recent longitudinal study connecting app-related activity to NSSI behaviors and thoughts found that greater engagement on this app was associated with decreased likelihood of NSSI thoughts and fewer intentions to injure within a week’s time, whereas posting triggering content was related to increased likelihood of both NSSI thoughts and behaviors [50]. However, to date, there has not been a test of this platform’s efficacy in improving outcomes relevant to NSSI recovery. This trial is designed to meet this need.

Given the lack of prior work testing the effects of publicly available web-based and mobile platforms as resources for NSSI recovery, we sought to provide preliminary evidence of a treatment effect pursuant to future research and larger-scale trials. The broad aims of this small-scale trial are to assess the shorter- and longer-term efficacy of using the peer support app in mitigating NSSI frequency and urges, increasing contact with informal support and interest in therapy, and improving attitudes toward professional help–seeking. We present several hypotheses and research questions related to these primary outcomes. Please note that the hypotheses and research questions presented in this manuscript are part of a larger preregistered set. Because of power constraints, we did not include planned tests of mediation and reduced the number of variables explored in some cases (eg, H2). In addition, the ordering of these hypotheses differs from the study preregistration to assist in the logical flow of the results.

Specifically, we hypothesized that participation on this app would be associated with improvements in NSSI outcomes, readiness to change, and attitudes and behaviors related to support and help-seeking. Our hypotheses were guided by theory and existing literature—largely on perceived effects of web-based communities (from the vantage point of individuals with lived NSSI experience) and prior empirical support for the role of social support in NSSI recovery:

- H1: Participation on the peer support app would lead to reductions in NSSI (i) frequency and (ii) urges, as well as increases in (iii) readiness to change compared with the control group.
- H2: Participation on the peer support app would lead to increases in (i) informal conversations, (ii) satisfaction derived from these conversations, (iii) interest in therapy, and (iv) improved attitudes toward formal help-seeking compared with the control group.

We pose an exploratory research question of secondary outcomes that may be associated with participation on a web-based peer support platform based on prior qualitative research:

- RQ1: Will participation on the peer support app lead to increases in (i) sense of belonging and (ii) social connectedness, as well as reductions in (iii) internalized stigma?

We also hypothesized a dose–response relationship among those in the peer support app treatment group, wherein greater engagement (sessions per week) would strengthen treatment effects:

- H3: There would be a dose–response relationship between app use and the magnitude and durability of the effect of use on NSSI (i) frequency and (ii) urges.

Given that the peer support app is a relatively lightweight and nonprofessional intervention, we explored the durability of the postintervention effects:

- RQ2: Will the effect of participation on the peer support app on NSSI (i) frequency and (ii) urges as well as (iii) readiness to change be maintained at 1-month and 2-month follow-up?

## Methods

### Trial Design and Procedure

This study was supported by a small pilot program grant and was intended to serve as a small-scale trial that would allow us to explore the feasibility and necessary parameters required for a larger outcome trial. All study procedures were approved by the institutional review board at our university and the trial was preregistered at the Open Science Foundation. This was a 2-arm randomized controlled trial. Participants in the treatment group were invited to use the peer support app platform for a duration of 8 weeks. They were instructed to engage with the platform (publish posts or comments) at least three times per week. Although the trial was not deemed to elevate participant risk and was, in fact, intended to reduce risk, we were careful to minimize the likelihood of participant discomfort through careful consideration of inclusion and exclusion criteria, using a platform with strong in-app user protections, regularly reminding participants of available resources, and following up on anyone who expressed discomfort through communication with the research team.

To inform our decision regarding the amount of engagement that would be appropriate for participants using the app, we conducted preliminary analysis on a large sample of existing app users. A latent profile analysis of 105,504 users who had been flagged by self-injury classifiers or had posted within the self-injury thread suggested that moderate in-app engagement was associated with 3.35 posts and comments (combined) per week. These analyses also suggested that a 6-8-week trial duration would be ideal because this was well within the range of natural use.

Participants in the control group received weekly psychoeducational materials regarding NSSI through email. Psychoeducation is commonly a component of digital mental health interventions [51-53] and was chosen as an appropriate control because it was not participative or interpersonal and could easily be delivered electronically. The decision to use an active versus waitlist control was largely to keep participants engaged through the intervention period. Both groups were asked to complete a survey at baseline and weekly for the duration of the intervention period (8 weeks) and at 1 month and 2 months after the intervention period.

### Participant Eligibility

Participants aged 16-25 years with current (within 3 months) and chronic (>6 episodes in the past year) NSSI history were eligible to participate in this study. The exclusion criteria included recent history of psychosis (>2 weeks’ institutionalization in the past year) or current suicidality (operationalized as suicidal thoughts or plans at baseline). Potential participants were screened in a web-based eligibility survey. Upon completion of this eligibility survey, all eligible participants received an email from the research team providing them with key information regarding their participation and a web-based consent document. Participants were randomly assigned to the treatment or control condition upon consent, using a random number generator to avoid bias. The consent document for participants in the treatment condition informed them that the research team would have access to their use data on the mobile app for the duration of the study and follow-up period. All participants then received a welcome email containing several videos explaining expectations per week, how to register for the platform (where applicable), and details regarding how and when they would receive compensation.

### Recruitment

Recruitment occurred through solicitations posted on (1) self-injury information clearing house websites and (2) through affiliated professional networks, social media outlets (such as Facebook or Twitter), and listservs, as well as (3) through the university recruitment system. Participants were eligible to receive a total of US $90 in the form of Amazon gift cards for completion of the study components (weekly surveys) throughout the study. Participants were compensated based on the number of weekly surveys they submitted. The trial ran from April 2019 to April 2020, with the last follow-up in June 2020.

### Outcome Measures

The primary outcome variables were assessed at each time point throughout the study—a total of 8 time points (week 1-8) were considered, with the addition of 2 time points (1 month and 2 months after the intervention period) in follow-up analysis. Baseline measures on primary outcomes were controlled for in their respective analysis, as were demographics: gender and country of origin. On the basis of high comorbidity among individuals who engage in NSSI and the potential for this to affect the engagement and efficacy of treatment [54,55], lifetime NSSI frequency (assessed with the NSSI Assessment Tool [NSSI-AT]; [56]) and mental health and trauma histories were also controlled for. Mental health history was assessed with a self-report checklist of 13 mental health conditions wherein participants were asked the following question: “To the best of your knowledge, have you ever suffered from any of the following?” Trauma history was assessed through the Stressful Life Events Screening Questionnaire [57]. Both variables were operationalized as counts of the number of mental health conditions or traumas (eg, death of a parent and sexual abuse) that participants reported at baseline (see Table 1 for details on participant characteristics).

### Primary Outcomes

#### NSSI Frequency

Participants completed a self-injury form checklist from the NSSI-AT [56]: “In the past week, have you ever done any of the following with the purpose of intentionally hurting yourself?” Response options were *Yes* or *No*. Participants who responded *Yes* were then asked the number of times they intentionally hurt themselves. Response options were on an 8-point scale, 0-7, with 0 reflecting no NSSI, 1 reflecting *Only once* and 7 reflecting *More than 50 times*.

#### NSSI Urges

Urges to self-injure were measured with two items adapted from the Alexian Brothers Urge to Self-Injure Scale [58]: “How often have you thought about injuring yourself in the last week?” and “How difficult was it to resist injuring yourself in the last week?” The first item was measured on a continuous scale from 1=*Never* to 100=*Nearly all of the time*, with a midpoint of *Sometimes (1-2 times per day or 5-10 times per week)*. The second item—“How difficult was it to resist injuring yourself in the last week?”—was measured on a 7-point scale ranging from 1=*Not at all difficult* to 7=*Was not able to resist*.

#### Readiness to Change

Readiness to change was assessed with the Readiness Ruler—a simple tool used to help patients visualize their readiness to change. Participants indicated where they fell regarding their readiness to change, confidence in their ability to change, and importance of change on a scale of 1=*Absolutely not true* to 10=*Absolutely true*. The items included: “Taking steps toward stopping self-injury is important to me,” “I am ready to take steps toward stopping self-injury,” and “I am confident I can take steps toward stopping self-injury.” This measure is typically used in clinical contexts, but its use in studies with constraints—such as field or lengthy surveys—has been suggested [59,60].

#### Contact With Informal Support

Informal support was operationalized as both (1) the number of conversations a participant reported having about self-injury and (2) the number of conversations that participants perceived as helpful. These were assessed through disclosure items from the NSSI-AT. Specifically, participants were asked if someone knew about their self-injury at baseline and if they had had a conversation about their self-injury in weekly surveys. If yes was selected, participants were asked to check boxes for the categories of people with whom they had had these conversations (eg, parent or guardian and friend). The number of boxes selected (or categories represented) were then summed weekly. If participants indicated that they had had a conversation with someone, they were also asked, “Have the conversations you’ve had with this person been helpful?” The response options were *Yes*, *No*, and *I don’t know*. As in the number-of-conversations measure, the number of helpful conversations was summed weekly.

#### Interest in Therapy

NSSI-AT Treatment Experiences items were used to assess interest in therapy. Participants responded to “How interested are you in attending therapy in the next month?” at baseline and weekly. The response options were assessed on a 5-point scale ranging from 1=*Not at all interested* to 5=*Very interested*.

#### Attitudes Toward Professional Help–Seeking

Attitudes toward professional help–seeking were assessed through the Attitudes Toward Seeking Professional Psychological Help Scale [61]. Participants were asked to rate the extent to which they agreed with 5 items meant to assess their attitudes toward help-seeking. These items were assessed on a 5-point scale ranging from 1=*Disagree* to 5=*Agree* (Cronbach α=.70).

### Secondary Variables

#### Internalized Stigma

Mental health stigma was measured through the Internalized Stigma of Mental Illness Scale [62]. This measure consists of 3 subscales (alienation, withdrawal, and stereotype) with 4 items each. Participants indicated the extent to which they agreed with statements on a 5-point Likert scale (from 1=*Strongly disagree* to 5=*Strongly agree*). Higher values indicate greater internalized stigma. All scales demonstrated acceptable factor structure—alienation: Cronbach α=.78, withdrawal: Cronbach α=.84, and stereotype: Cronbach α=.70.

#### Sense of Belonging

Sense of belonging was measured through the belonging subscale of the short form version of the Interpersonal Support Evaluation checklist [63]. Participants rated the extent to which they agreed with 4 statements on a 7-point scale from 1=*Strongly disagree* to 7=*Strongly agree*. Higher values indicate a greater sense of belonging (Cronbach α=.75).

#### Social Connectedness

Participants responded to the Social Connectedness Scale developed by Lee and Robbins [64] and rated the extent to which they agreed with 8 statements reflecting their sense of social connection on a 7-point scale from 1=*Strongly disagree* to 7=*Strongly agree*. Factor structure was acceptable (Cronbach α=.88).

#### Mobile App Activity

Data on participants’ mobile app activity were supplied with license from the platform and consent from participants. These data included the number of posts and comments participants published weekly over the course of the trial and follow-up periods and were used for a dose–response analysis.

### Statistical Analysis

Primary analyses were run on an intention-to-treat basis, with all participants randomized regardless of level of adherence. The relationship between survey completion as a continuous variable and demographics (gender, age, and region), indicators of mental health severity that may affect one’s ability to engage with the intervention (mental health diagnosis and trauma history), and attitudinal and motivational factors (eg, readiness to change, confidence in change, and importance of change) was also investigated through 1-way analysis of variance, where these predictors were independently regressed on survey completion. Missingness was not related to any of these variables. Finally, because some participants completed their week 8 and follow-up surveys during the COVID-19 pandemic, we ran parallel analyses that included and excluded data points that fell within the period in which most countries and states had formalized stay-at-home orders. Only 6.9% (9/131) of the participants completed their final intervention week (week 8) during the COVID-19 pandemic period (treatment=2 and control=7). Given this small sample, we observed no significant differences in the main analyses.

Linear mixed models (LMMs) were used to examine each of our primary outcome variables. Several estimators (maximum likelihood and restricted maximum likelihood) and covariance structures (first-order autoregressive process, compound symmetry, and unstructured) were compared before arriving at a combination that best fit our data: a maximum likelihood estimator with a first-order autoregressive covariance structure. All models included a random intercept for participant, fixed effects of condition, time point, a condition by time point interaction term, and relevant covariates. Models controlled for demographics (gender and country of origin), mental health history (trauma, mental health diagnoses, and lifetime NSSI), and the primary outcome variable at baseline. When the time point by condition interaction effect was not significant, it was removed from the final model before interpreting significant main effects. All analyses were performed using SPSS software (versions 25 and 27; IBM Corp).

## Results

### Participant Characteristics

**Figure 1:**
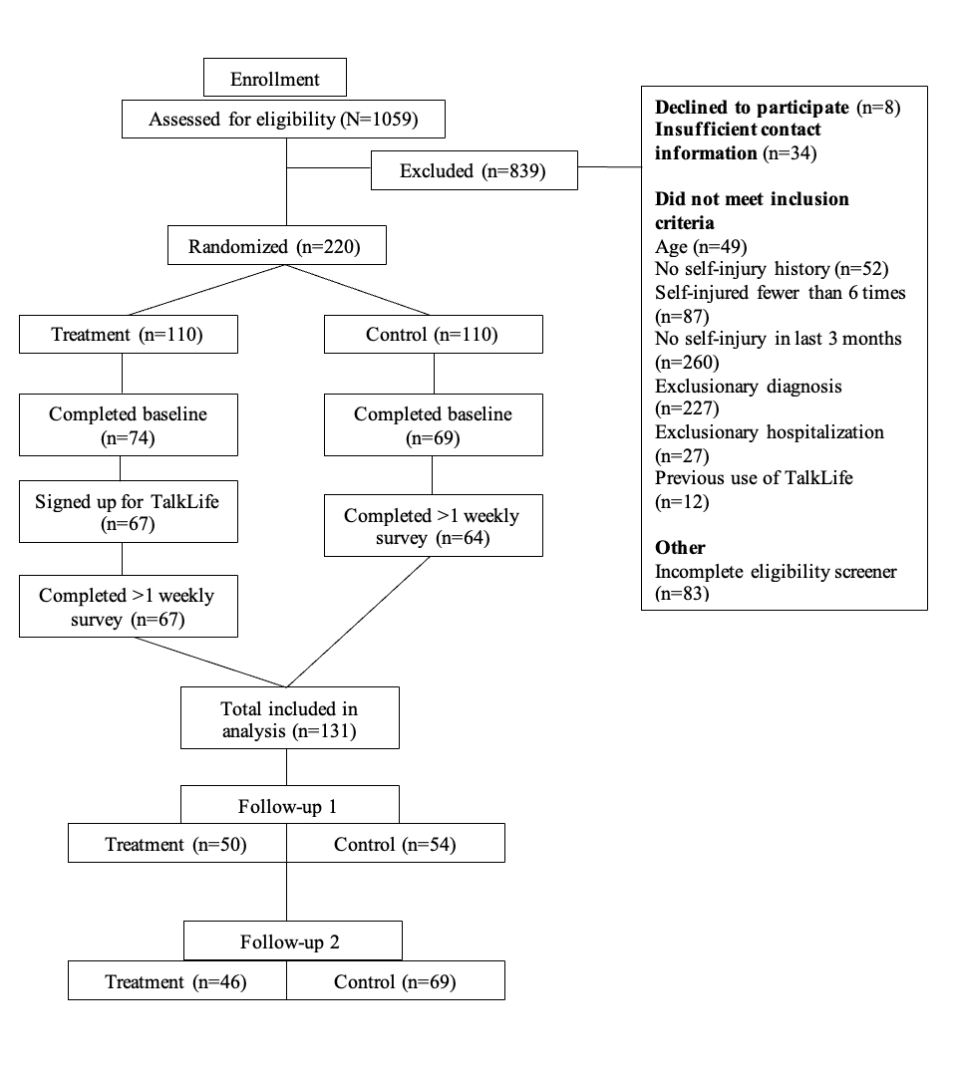
Flow of participants.

A total of 131 participants were randomized into treatment and control conditions and completed baseline surveys. The flow of participants through the study is depicted in Figure 1. Participants completed a mean of 6.48 (SD 2.36) of the 8 total surveys during the treatment period on average. Completion rates were not statistically different by condition: t_129_=–0.65; *P=*.51; Cohen *d*=–0.11 (intervention: mean 6.36, SD 2.45; control: mean 6.63, SD 2.24). In terms of engagement with the app among those in the treatment arm, the mean number of posts and comments published per week was 3.13 (SD 2.46), with an average of 8.43 (SD 9.36) sessions per week over the study period.

Table 1 depicts the basic demographics, comorbidities, and key variables in these groups at baseline. The groups differed significantly at baseline on self-reported interest in therapy (P=.01), attitudes toward professional help–seeking (P<.001), and social connectedness (P<.001).

**Table 1:** Participant characteristics and key variables at baseline based on condition (N=131).

					Full sample (N=131)		Treatment (n=67)		Control (n=64)		Significance test						P value	
											*t* test (*df*)			Chi-square (*df*)				
**Demographics**																		
	Age (years), mean (SD)			20.32 (2.52)		20.04 (2.24)		20.61 (2.76)		–1.29 (129)			N/A^a^			.20		
	**Gender, n (%)**											N/A			2.4 (3)			.49
		Male		24 (18.3)		13 (19.4)		11 (17.2)										
		Female		89 (67.9)		42 (62.7)		47 (73.4)										
		Nonbinary		15 (11.5)		10 (14.9)		5 (7.8)										
		Other		3 (2.3)		2 (3)		1 (1.6)										
	**Region, n (%)**											N/A			0.5 (2)			.78
		North America		82 (62.6)		40 (59.7)		42 (65.6)										
		European Union		24 (18.3)		13 (19.4)		11 (17.2)										
		United Kingdom		25 (19.1)		14 (20.9)		11 (17.2)										
**NSSI^b^ characteristics**																		
	**Age at first** **NSSI (years), n (%)**											N/A			7.6 (4)			.11
		≤10		19 (14.5)		11 (16.4)		8 (12.5)										
		11-12		35 (26.7)		11 (16.4)		24 (37.5)										
		13-14		43 (32.8)		25 (37.3)		18 (28.1)										
		15-16		18 (13.7)		10 (14.9)		8 (12.5)										
		≥17		16 (12.2)		10 (14.9)		6 (9.4)										
	**NSSI frequency^c^, n (%)**											N/A			1.3 (2)			.53
			2-3 times per week	112 (85.7)		59 (88.1)		53 (82.8)										
			1 time per week	10 (7.6)		5 (7.5)		5 (7.8)										
			1-3 times per month or less	9 (6.9)		3 (4.5)		6 (9.4)										
	**Lifetime NSSI (NSSI total)^d^**											N/A			1.9 (2)			.40
			4-20 times, n (%)	8 (6.1)		3 (4.5)		5 (7.8)										
			21-50 times, n (%)	28 (21.4)		12 (17.9)		16 (25)										
			More than 50 times, n (%)	95 (72.5)		52 (77.6)		43 (67.2)										
	Urges: thoughts, mean (SD)			50.43 (23.74)		50.91 (26.81)		49.93 (20.24)		0.24 (130)			N/A			.81		
	Urges: difficulty resisting, mean (SD)			4.52 (1.46)		4.37 (1.48)		4.67 (1.43)		–1.17 (129)			N/A			.24		
**Informal and formal support**																		
	Conversations (yes or no), n (%)			112 (85.5)		60 (89.6)		52 (81.3)		N/A			1.8 (1)			.18		
	Number of roles, mean (SD)			4.26 (2.17)		4.07 (2.07)		4.48 (2.27)		0.32 (110)			N/A			.32		
	Number of helpful conversations with roles, mean (SD)			2.03 (1.42)		2.03 (1.46)		2.01 (1.39)		0.05 (111)			N/A			.96		
	Therapy (yes or no), n (%)			105 (80.2)		56 (83.6)		59 (76.6)		N/A			1.0 (1)			.38		
	NSSI in therapy (yes or no)^e^, n (%)			90 (85.7)		49 (73.1)		41 (83.7)		N/A			0.3 (1)			.58		
	Helpfulness of therapy overall (1=not at all helpful; 10=very helpful), mean (SD)			5.89 (2.51)		6.16 (2.59)		5.60 (2.41)		1.13 (102)			N/A			.26		
	Helpfulness of therapy in stopping NSSI (1=not at all helpful; 5=very helpful), mean (SD)			2.52 (1.43)		2.35 (1.14)		2.18 (1.34)		0.76 (94)			N/A			.45		
	Interest in therapy (scale: 1-5), mean (SD)			2.84 (1.77)		1.63 (1.43)		3.04 (1.74)		–2.53 (72)			N/A			.01		
	ATSPPH^f^ (scale: 5-35), mean (SD)			21.09 (7.36)		25.53 (5.70)		16.45 (5.91)		8.95 (129)			N/A			<.001		
**Other characteristics, mean (SD)**															N/A			
	Importance of change			7.14 (2.49)		7.05 (2.54)		7.22 (2.44)		–0.36 (129)						.72		
	Readiness to change			6.64 (2.68)		6.49 (2.78)		6.79 (2.58)		–0.65 (129)						.52		
	Confidence in change			5.68 (2.59)		5.51 (2.52)		5.85 (2.67)		–0.78 (129)						.44		
	Internalized stigma, total			2.88 (0.61)		2.89 (0.523)		2.86 (0.70)		0.29 (129)						.83		
	Stigma: stereotype			2.20 (0.72)		2.09 (0.633)		2.33 (0.79)		–1.94 (129)						.05		
	Stigma: alienation			3.59 (0.74)		3.68 (0.668)		3.49 (0.80)		1.45 (129)						.15		
	Stigma: withdrawal			2.83 (0.93)		2.89 (0.843)		2.77 (1.01)		0.78 (129)						.44		
	Belongingness			14.40 (5.72)		14.60 (6.03)		14.19 (5.43)		0.41 (129)						.68		
	Social connectedness			27.56 (10.82)		31.21 (10.92)		23.73 (9.37)		4.19 (129)						<.001		
	Trauma history (total traumatic events)			3.52 (2.01)		3.28 (1.93)		3.77 (2.08)		–1.37 (129)						.17		
	Mental health history (total mental health conditions)			3.24 (1.82)		3.24 (1.83)		3.23 (1.92)		0.014 (129)						.99		

^a^N/A: not applicable.

^b^NSSI: nonsuicidal self-injury.

^c^The last 2 categories were collapsed for nonsuicidal self-injury frequency 1-3 times per month and 1 time every month because of low cell sizes.

^d^The first 3 categories were collapsed for lifetime nonsuicidal self-injury (4-5 times, 6-10 times, and 11-20 times) because of low cell sizes.

^e^Of those who reported attending therapy (n=105).

^f^ATSPPH: Attitudes Toward Seeking Professional Psychological Help.

### Primary Outcomes

#### Overview

No significant time point by condition effects were observed in our analysis of primary outcomes, suggesting that patterns of change were not linear or equivalent across groups (see Multimedia Appendix 1 for line graphs of primary outcomes over the course of the trial and follow-up periods). We thus report on the results of LMM models after the interaction effect was removed. Condition effects, marginal means, and effect sizes are presented in Table 2.

**Table 2:** Differences in outcomes by condition^a^.

			Full sample, mean (SE)	Treatment, mean (SE)	Control, mean (SE)	*F* test (*df*)	P value	*η* ^2^ ^b^
**Primary outcomes^c^**								
	**NSSI^d^**							
		NSSI frequency	1.46 (0.17)	1.30 (0.18)	1.62 (0.18)	5.78 (1,129.91)	.02	0.02
		Urges: thoughts	44.53 (3.79)	42.69 (4.06)	46.37 (4.09)	1.53 (1,124.63)	.22	0.001
		Urges: difficulty resisting	3.36 (0.24)	3.31 (0.26)	3.42 (0.26)	0.314 (1,121.39)	.58	0.001
	**Readiness to change**							
		Importance of change	7.80 (0.35)	8.03 (0.37)	7.57 (0.38)	3.21 (1,112.70)	.08	0.006
		Readiness to change	6.69 (0.39)	6.96 (0.43)	6.42 (0.42)	2.91 (1,126.20)	.09	0.01
		Confidence in change	5.97 (0.38)	6.28 (0.41)	5.67 (0.41)	4.27 (1,127.33)	.04	0.02
	**Informal and formal support**							
		Weekly informal conversations	0.31 (0.12)	0.28 (0.13)	0.33 (0.13)	0.35 (1,107.76)	.55	0.05
		Satisfaction derived from weekly conversations	0.72 (0.24)	0.73 (0.26)	0.71 (0.26)	0.02 (1,88.54)	.88	0.06
		Interest in therapy (scale: 1-5)	2.93 (0.32)	2.93 (0.38)	2.94 (0.32)	0.002 (1,63.31)	.97	0.03
		Attitudes toward professional help–seeking (scale: 5-35)	25.95 (1.36)	26.34 (1.48)	25.56 (1.53)	0.36 (1,127.06)	.55	0.01
**Secondary outcomes**								
	Internalized stigma: stereotype		2.27 (0.13)	2.30 (0.14)	2.23 (0.14)	0.39 (1,129.49)	.53	0.01
	Internalized stigma: alienation		3.51 (0.16)	3.40 (0.17)	3.62 (0.18)	2.94 (1,128.81)	.09	0.08
	Internalized stigma: withdrawal		2.62 (0.16)	2.55 (0.16)	2.69 (0.17)	1.68 (1,122.39)	.19	0.01
	Sense of belonging		15.19 (0.71)	15.91 (0.75)	14.44 (0.77)	7.45 (1,128.83)	.007	0.06
	Social connectedness		29.21 (1.87)	28.65 (2.08)	29.77 (2.01)	0.47 (1,121.12)	.49	0.02

^a^All means reflect estimated marginal means from adjusted models.

^b^For effect size *η*^2^*,* 0.01 corresponds to a small effect, 0.06 corresponds to a medium effect, and 0.14 corresponds to a large effect [65].

^c^Covariates include gender, region, trauma and mental health history, nonsuicidal self-injury frequency and lifetime nonsuicidal self-injury, and tested outcome at baseline. In addition, models control for time point and random effect of participant.

^d^NSSI: nonsuicidal self-injury.

#### NSSI Frequency and Urges

Significant effects were observed for NSSI frequency (H1i) such that on average, participants in the peer support app condition injured themselves less over the course of the study (mean 1.30, SE 0.18) than participants in the control condition (mean 1.62, SE 0.18; P=.02; H1i). We did not observe any differences by condition for NSSI urges (H1ii) (see Table 2 for full results).

#### Readiness to Change

A significant effect of treatment on confidence in one’s ability to change NSSI behaviors was observed (H1iii). Specifically, participants using the peer support app reported greater confidence in their ability to change their NSSI behavior (mean 6.28, SE 0.41) compared with control participants (mean 5.67, SE 0.41; P=.04). No significant effects were found for importance of changing NSSI behavior or readiness to change (see Table 2 for full results).

#### Informal Support, Interest in Therapy, and Attitudes Toward Professional Help–Seeking

No significant differences were evidenced between the groups or across time for any informal support or help-seeking outcomes (H2) including (i) informal conversations, (ii) satisfaction derived from these conversations, (iii) interest in therapy, and (iv) attitudes toward professional help–seeking (see Table 2 for full results).

### Secondary Outcomes

Exploratory analyses of internalized stigma, sense of belonging, and social connectedness as secondary variables (RQ1) revealed a significant condition effect for sense of belonging such that participants in the peer support app group reported greater sense of belonging (mean 15.91, SE 0.75) compared with those in the control group (mean 14.44, SE 0.77; P=.007). Time point was also significant for sense of belonging (P<.001), but the interaction between time point and condition did not reach significance (*F*_7,732.87_=1.89; P=.07). There were no other significant effects for internalized stigma or social connectedness (see Table 2 for full results).

### Dose–Response Relationship

To explore the potential for a dose–response relationship between app use and NSSI frequency and urges, log data from the platform were used (H3). Specifically, all participants’ posts and comments were summed at the week level. Several data points were observed at 3 times the IQR (21 data points from 10 participants). After inspection of participant trends and confirming normality in their responses on other study measures, they were deemed outliers. Winsorizing was selected to reduce the pull of these significant outliers while retaining their data [66].

The dose variable was entered as a predictor in the main LMMs, as described previously. No significant effects of dose were found for NSSI frequency (H3i; *F*_1,403.96_=2.17; P=.14; *η*^2^=0.04) or urges (H3ii; NSSI thoughts: *F*_1,390.14_=1.02; P=.31; *η*^2^=0.002; difficulty resisting: *F*_1,309.36_=0.39; P=.53; *η*^2^=0.005). A test of sensitivity was run by comparing the results with the raw, nonwinsorized values, and the results remained insignificant: NSSI frequency (H3i): *F*_1,292.19_=0.79; P=.38; *η*^2^=0.03; NSSI thoughts (H3ii): *F*_1,258.37_=0.02; P=.89; *η*^2^=0.003; and difficulty resisting (H2ii): *F*_1,157.75_=0.44; P=.51; *η*^2^=0.005.

### Follow-Up Analyses

Follow-up analyses (RQ2i-iii) were conducted to explore the durability of effects at 1 month and 2 months after the intervention period. We ran 2 LMMs that included all data during the intervention period (weeks 1-8) in addition to data at first follow-up (1 month) or all data during the intervention period in addition to data at first and second follow-up (1 month and 2 months). The results showed a decay in intervention effects at both follow-up periods. Given the similarities across both follow-up periods, we report statistics for the 2-month follow-up here.

At 2 months after the intervention period, the condition effect of NSSI frequency (H1i) remained significant (*F*_1,130.16_=5.49; P=.02; *η*^2^=0.02) such that participants using the peer support app continued to report lower mean NSSI frequency (mean 1.24, SE 0.17) compared with the control participants (mean 1.54, SE 0.18). The effect of condition on NSSI urges (RQ2ii) remained insignificant (thoughts: *F*_1,117.29_=1.62; P=.21; *η*^2^=0.001; difficulty resisting: *F*_1,121.99_=0.98; P=.32; *η*^2^=0.01). In terms of readiness to change (RQ2iii), the effect on confidence in ability to change was not sustained (*F*_1,130.32_=3.63; P=.06; *η*^2^=0.02), and importance of change (*F*_1,133.83_=1.59; P=.21; *η*^2^=0.02) and readiness to change (*F*_1,128.37_=2.13; P=.15; *η*^2^=0.01) continued to be insignificant.

## Discussion

### Principal Findings

Overall, our findings suggest that when compared with provision of web-based psychoeducational materials, use of the peer support platform was associated with reduced NSSI frequency over the course of the 8-week study period (H1i). Significance was sustained at both 1- and 2-month follow-up periods (RQ2i), with slight reductions in the magnitude of the effect at each reporting period. We also found a treatment effect for confidence in one’s ability to change NSSI behaviors. Participants in the treatment group reported greater confidence in their ability to change behaviors over the course of the study (H1iii) compared with those receiving psychoeducational materials. However, this effect was not sustained at the follow-up periods (RQ2iii).

Given that effect sizes were small for both treatment effects and this study was not fully powered to detect small effects, the results should be interpreted with appropriate caution. Digital interventions targeting mental health outcomes are often characterized by small to moderate treatment effects [36,67,68], and even highly structured and time-intensive clinical treatments for NSSI result in small treatment effects [69]. Although trials of web-based support groups and peer-to-peer interventions vary in their efficacy on mental health outcomes, it is not uncommon for effect sizes to be small to moderate in powered trials [70-74]. This is a factor worth considering in future research because rolling recruitment for this study endured for more than one year. Notwithstanding these limitations, evidence of sustained effects of low-intensity engagement in the treatment group over 2 months is promising. These findings suggest the potential of lightweight interventions—such as peer support apps—as among the resources that may benefit young people engaging in NSSI and as worthy of future investigation.

Counter to our expectations, there were no treatment effects on NSSI urges, contact with informal support, interest in therapy, or attitudes toward professional help–seeking. Urges are an important clinical feature of NSSI; however, not all individuals who engage in NSSI report urges [58], and research suggests that those with more severe NSSI behaviors are more likely to report urges [75]. Future work may wish to consider other measures that may be more sensitive to change across subgroups.

Use of the peer support platform was not associated with increased offline conversations regarding their NSSI behaviors. Although models of web-based disclosure [76-78] often assume that increased comfort in making disclosures and reductions in stigma can result from web-based discourse and supportive exchange and subsequently prompt offline, in-person disclosures, there is not strong empirical support for this in the NSSI literature. In fact, one of the noted risks to web-based communication regarding NSSI is the potential for overidentification with the community and the potential for this to stunt alternative help-seeking [79,80]. Future research should follow up on this relationship through the inclusion of behavioral and attitudinal measures. Exploring the addition of specific guidance or interventions aimed at broadening help-seeking knowledge and increasing intentions to seek help, within these peer support spaces, is another worthy line for future work. We also feel the need to acknowledge the potential limitations of the measurements used to assess this construct and the limited variability we observed within and across participants. Informal conversations were computed by summing the number of roles (eg, parents, coaches, and peers) reflected in the weekly conversations regarding NSSI. Although this measure captures the types of individuals with whom the participants communicated over the course of the study, it was not capable of capturing who initiated the conversation (eg, self or other), the quantity of disclosures over time (eg, the number of friends), or the quality of conversations that ensued after disclosure. Each of these aspects of informal conversations and disclosure are important to consider in future work.

There was no evidence of a dose–response relationship in app use on any of the NSSI outcomes. Participants were instructed to engage on the platform at least three times (eg, publish 3 posts or comments) weekly. Although all participants engaged at this level for at least one week during the trial, we note variation in individual engagement across weeks. The mean number of posts and comments per week was 3.13 (SD 2.46), with an average of 8.43 (SD 9.36) sessions (log-ins) per week over the study period. The decision to prescribe 3 times of participative use per week was made to ensure that there was meaningful engagement beyond scrolling and based on past work suggesting that active use is more beneficial than passive use [81,82]. The fact that so many participants engaged at the prescribed level suggests that it is an appropriate baseline for future work. However, the range of this value also suggests that that it may have diverged from what would have been natural or normal use patterns for some. A lower threshold of engaged use or natural nonprescribed use may be warranted in future studies.

In terms of secondary variables, we did not find significant differences between the groups on internalized stigma or social connectedness; however, we did note a significant treatment effect for sense of belonging. Participants using the platform reported higher levels of belonging compared with the control group, and changes in sense of belonging were also trending toward significance over time. These findings are largely in line with research documenting sense of belonging and reduced loneliness as benefits of engagement in web-based communities [5,10]. These findings also align with open-ended user experience data that were collected as part of the week 8 survey in this trial, wherein the treatment group reported feeling less alone and expressed that a sense of shared experience as well as the destigmatizing nature of the exchanges were among the positive qualities of the platform (Kruzan et al, unpublished data, 2022). In brief, the participants’ qualitative responses showed a pattern of stronger positive and negative associations with the peer support platform compared with the participants in the control or psychoeducation condition who largely reported positive experiences. This dual harmful and helpful nature of the peer support app indicates that the relationship between app use and NSSI behaviors may not be as straightforward as our quantitative results suggest. For example, some participants suggested that the peer support app would have been helpful if they had been at a different (earlier) stage in their recovery process, underscoring the need to consider both readiness to change and stage of change in tailoring interventions, even in app-based settings [20,83]. We speculate that the differences observed among the participant outcomes reported in this paper and the self-reported experience may be in part due to the relative complexity and dynamic nature of interacting on a platform with other humans and the static quality of the psychoeducational materials.

Another lens that may explain some of the observed treatment effects but which was not explored in this study is social comparison. Prior work suggests that social comparison processes can play a negative role on peer-to-peer support platforms such that exposure to graphic content can trigger young people and drive competitiveness by making them feel as though their own NSSI behavior is not *severe* enough [25,26,84]. Less attention has been given to the possibility that certain comparisons (eg, downward social comparisons) may have a positive impact on NSSI by drawing individuals’ attention to the progress that they have made in their recovery, or by bringing increased awareness to current behaviors for those ready to contemplate change [12]. Engaging on a peer support platform and being exposed to NSSI content may uniquely position participants to see the need for change and to feel capable of changing with the support of others. In this sense, exposure to negative and graphic content may be an additional motivator influencing both readiness and frequency over the course of the study. In any case, the possibility of complex interactions between readiness to change and social comparison processes on these platforms should be further explored. Follow-up work is needed to disentangle the specific qualities of web-based spaces that may lead to incongruence in one’s subjective experience of the platform and outcome data.

Finally, although this was a small-scale trial with power limitations, we note that some of the small effects observed may be because the psychoeducational materials provided to the control participants were also regarded as efficacious (Kruzan et al, unpublished data, 2022) and seemed to exert some positive influence on several key outcome variables in this study (see Multimedia Appendix 1 for line graphs). Understanding the intervention utility of psychoeducational materials merits further empirical investigation because many publicly available web-based communities for NSSI do not currently include active psychoeducational components, but they may be a relatively easy and cost-effective addition for increasing access to such resources. Offering psychoeducational materials directly from a platform, either as a static resource or through a prompt (such as email), with some regularity may also reduce common motivational or attitudinal barriers to seeking such information independently. The findings from this trial also highlight a need to better understand the interaction of time and both study conditions. Although there were overall treatment effects or positive trends for both conditions, there were no meaningful linear trends from time point to time point, suggesting that the effects were not accrued through accumulation over time.

### Limitations

When interpreting the results, several limitations should be considered. First, the findings from this trial should be interpreted with appropriate caution, given that the study is underpowered to detect small effect sizes. There were few prior studies upon which we could base a priori assumptions and necessary parameters for sample size. Our study contributes some of these parameters for use in future research. Second, given the length of the trial and level of involvement requested of the participants, our sample may have been more motivated to engage in research (and thus with the intervention) than the average individual engaging in NSSI. Furthermore, we prescribed use of the platform, but natural use patterns may differ and this could affect outcomes. Future trials may wish to compare natural use to prescribed use. In addition, although we see an effect of app use on NSSI behavior, we were unable to explore possible mediators in this study. Future work should explore which elements of the experience on the platform drive the observed effect on NSSI frequency. Our findings suggest that sense of belonging may play an important role in this relationship. This trial did not control for a variety of other factors that may influence individuals’ NSSI behaviors over time, such as user expectancies, natural periodicity or the cyclical nature of NSSI, and other study procedures. We also note that our exclusion criteria affect the generalizability of our findings to individuals with more severe suicidality. Finally, although the need for this trial was in part informed by our prior work on the importance of social support in NSSI recovery [20], we did not directly involve individuals with lived experience in the trial design and must note this as a limitation and an important addition to future work.

### Conclusions

To date, there are few studies that have formally explored the role of web-based peer support in reducing NSSI behaviors and other factors that may support NSSI recovery. The prevalence of NSSI among young people and the tendency for them to disclose it and seek help in web-based spaces such as mobile apps and social media highlight a need for research exploring efficacy and disentangling key mechanisms. This small-scale trial explored the potential efficacy of a mobile peer support app in reducing NSSI behaviors and urges, increasing readiness to change, and increasing contact with support. Although we found only small effects of the platform on NSSI frequency and confidence in one’s ability to change NSSI behaviors over the course of the study, we did find evidence for increased supportive conversations and interest in therapy, as well as improved attitudes toward help-seeking behaviors. Furthermore, we found suggestive evidence that sense of belongingness may play a critical role in benefits derived from platform use. Future work investigating the key mechanisms underlying the efficacy of this app and other platforms where individuals exchange peer support in reducing NSSI behaviors through fully powered randomized controlled trials is warranted.

## Appendix

Multimedia Appendix 1 Line graphs of primary outcomes over time.

Multimedia Appendix 2 CONSORT-eHEALTH checklist (V 1.6.1).

## Abbreviations

CBT: cognitive behavioral therapy
LMM: linear mixed model
NSSI: nonsuicidal self-injury
NSSI-AT: Nonsuicidal Self-injury Assessment Tool
