# Supplementary material for: Use of a Mobile Peer Support App Among Young People With Nonsuicidal Self-injury: Small-scale Randomized Controlled Trial
Source: JMIR Form Res. 2022 Jan 10;6(1):e26526. doi: 10.2196/26526 (PMC8787664; doi:10.2196/26526)
Supplement: Multimedia Appendix 1 [file formative_v6i1e26526_app1.pdf]

Supplemental materials

Line graphs of primary outcomes over time

NSSI frequency

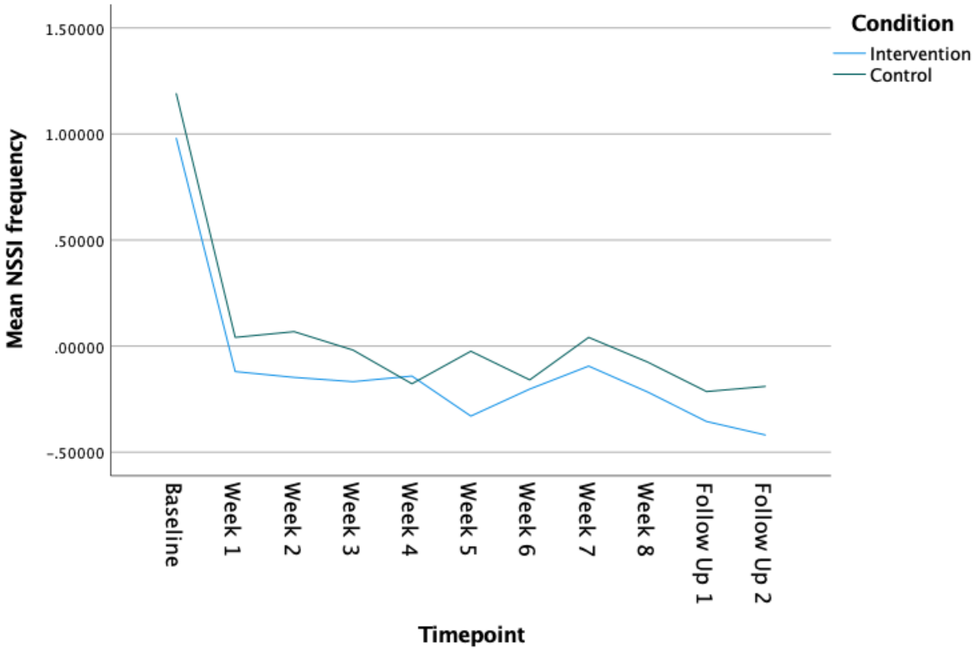

NSSI urges

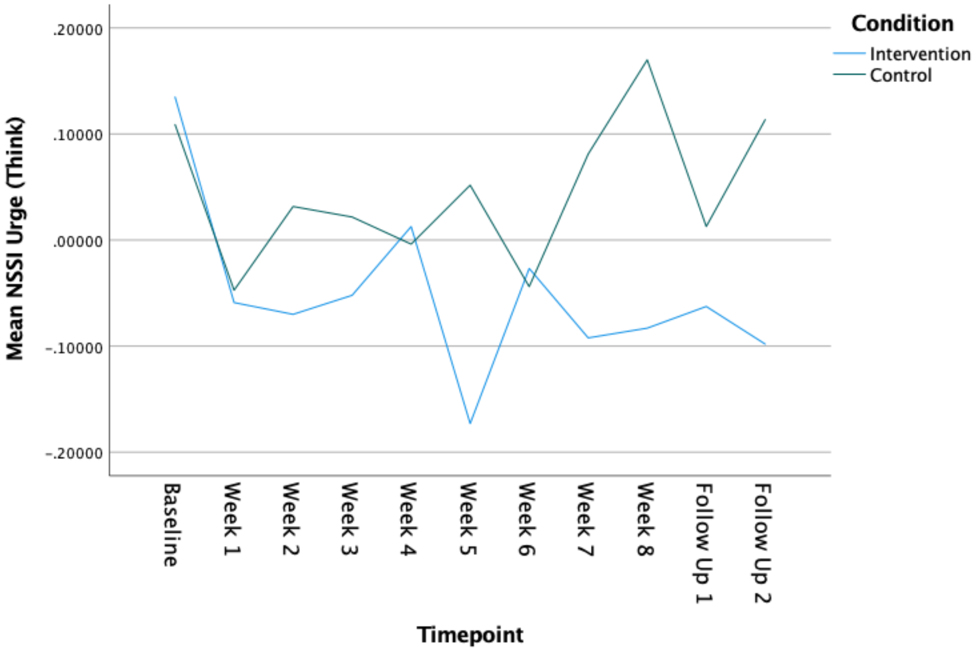

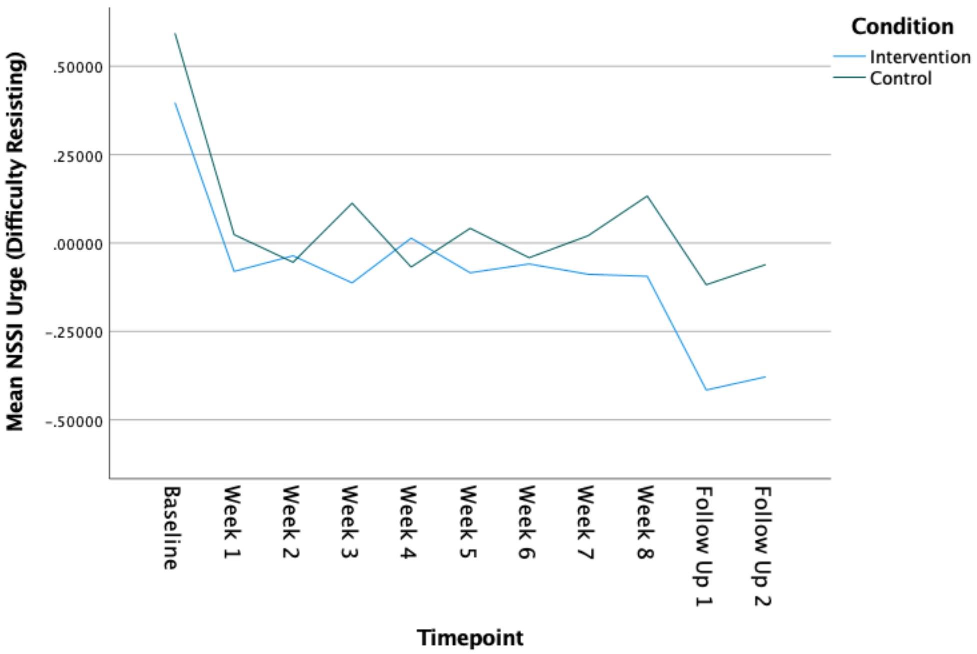

Importance of change

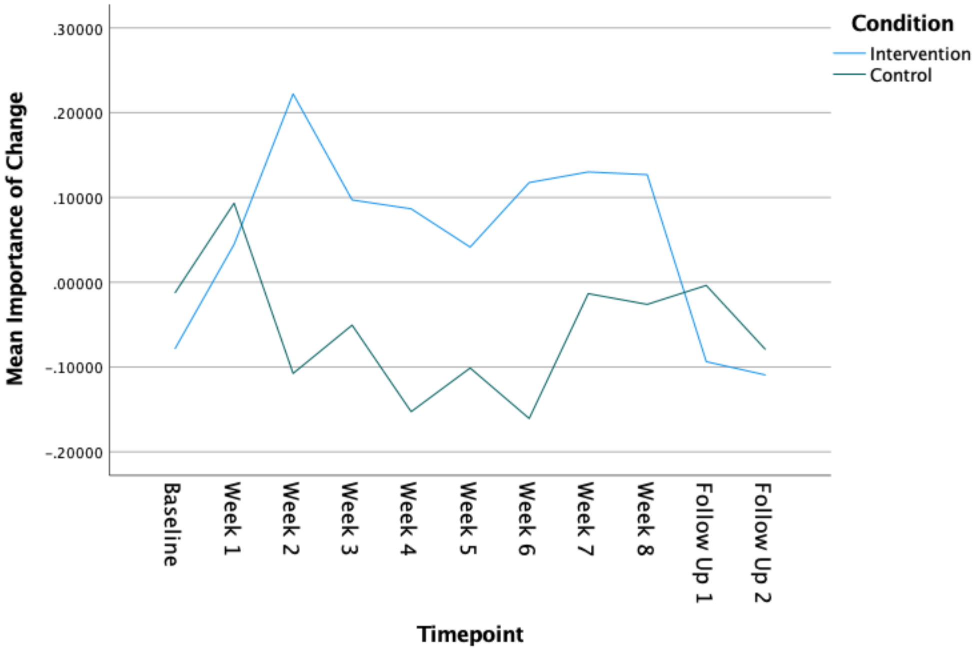

Readiness to change

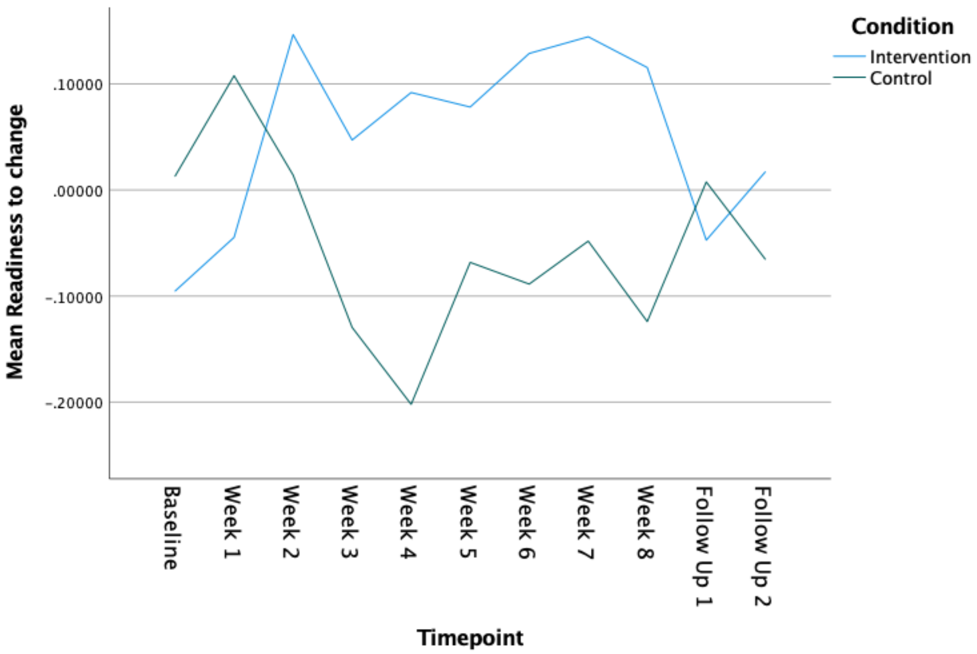

Confidence in one's ability to change

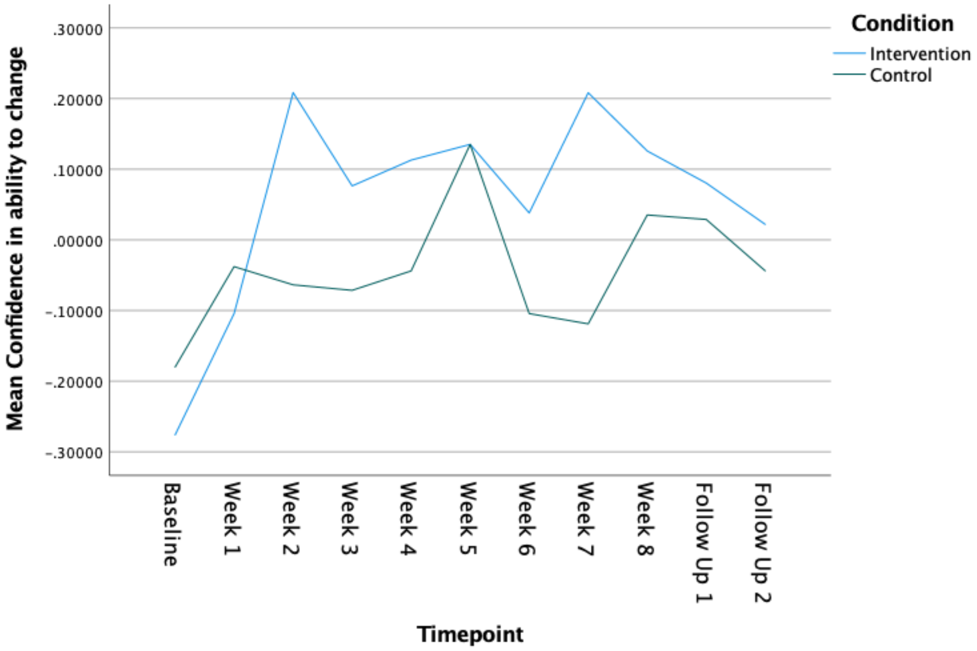

Informal conversations

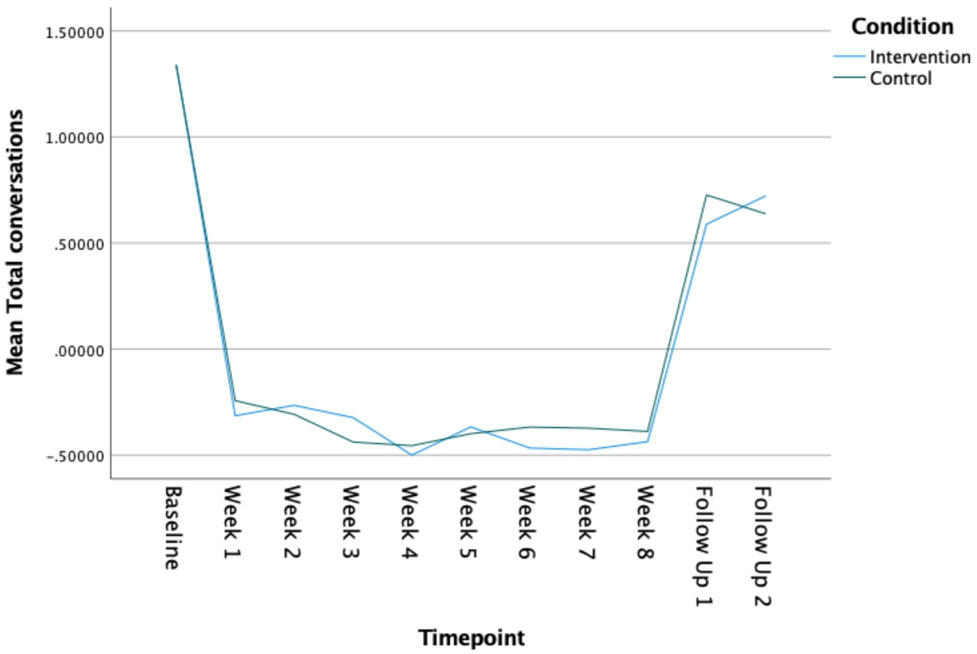

Satisfaction with conversations

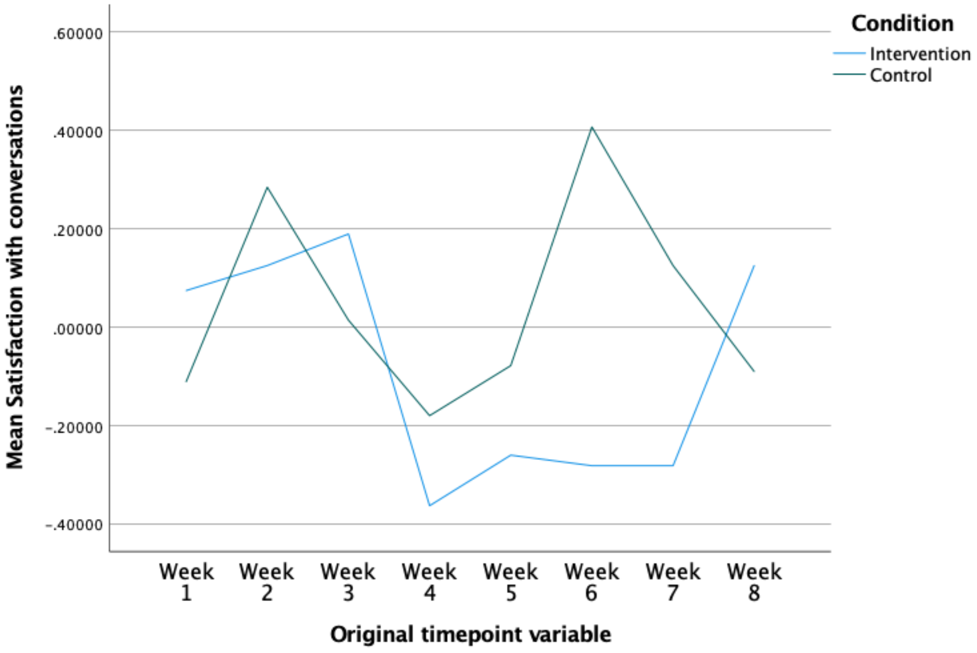

Attitudes towards professional help-seeking

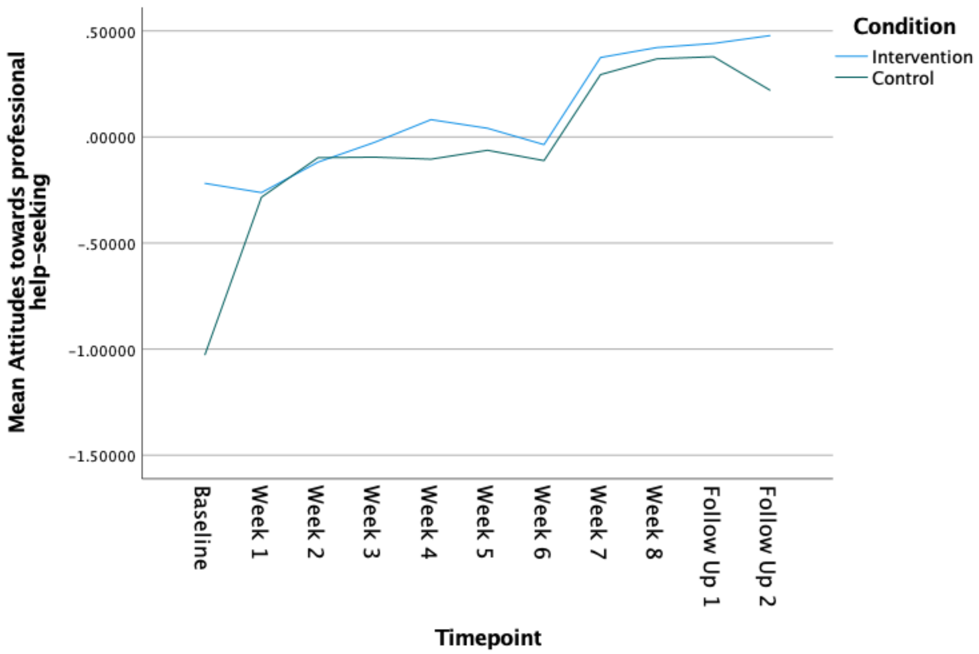

Interest in therapy

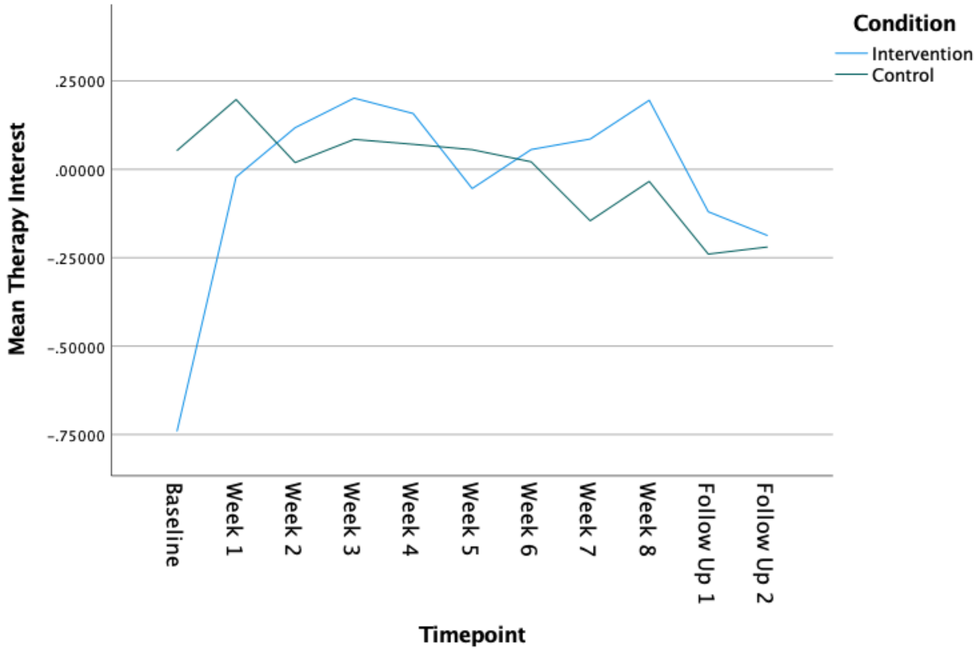

Secondary outcomes / mechanisms

Sense of belonging

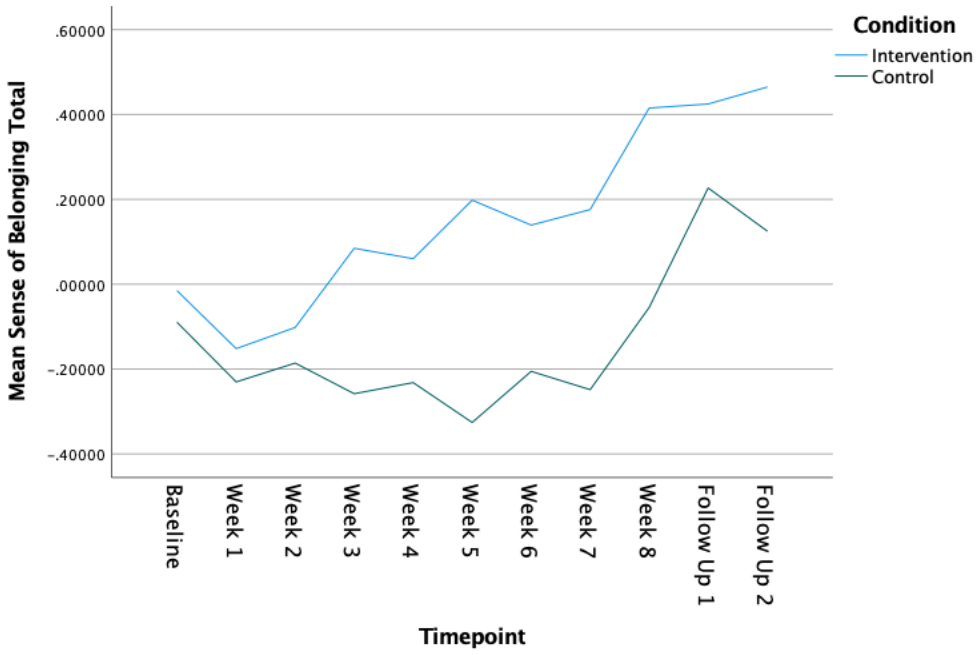

Stigma Alienation

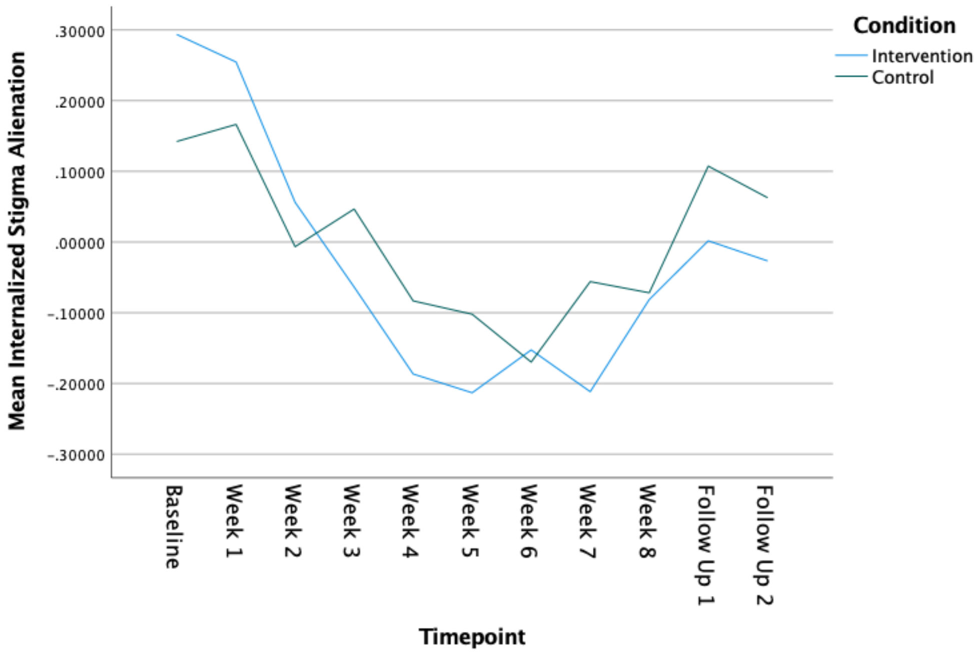

Stigma Withdrawal

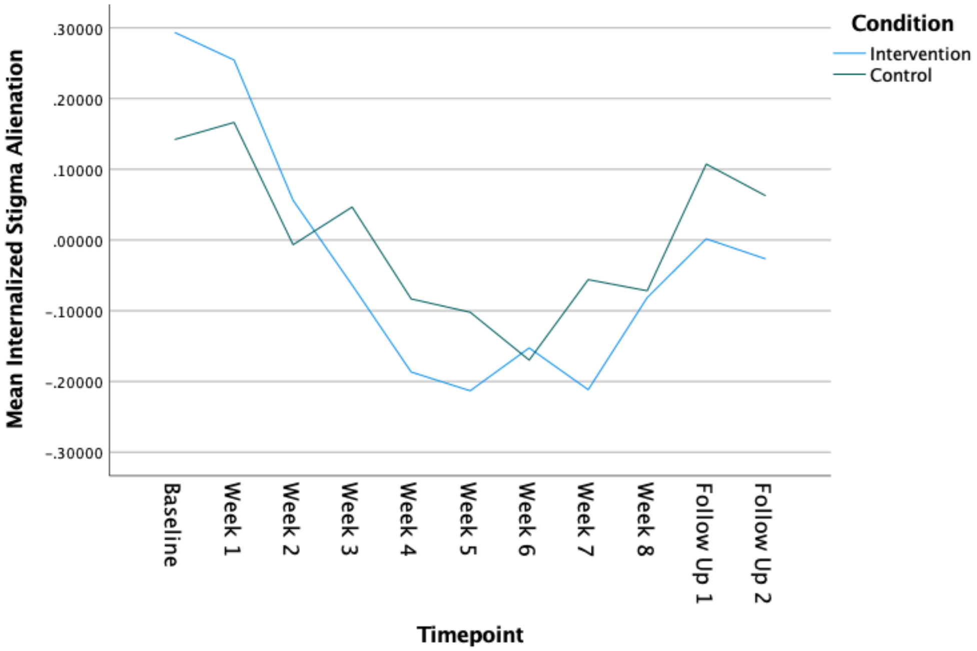

Stigma Stereotype

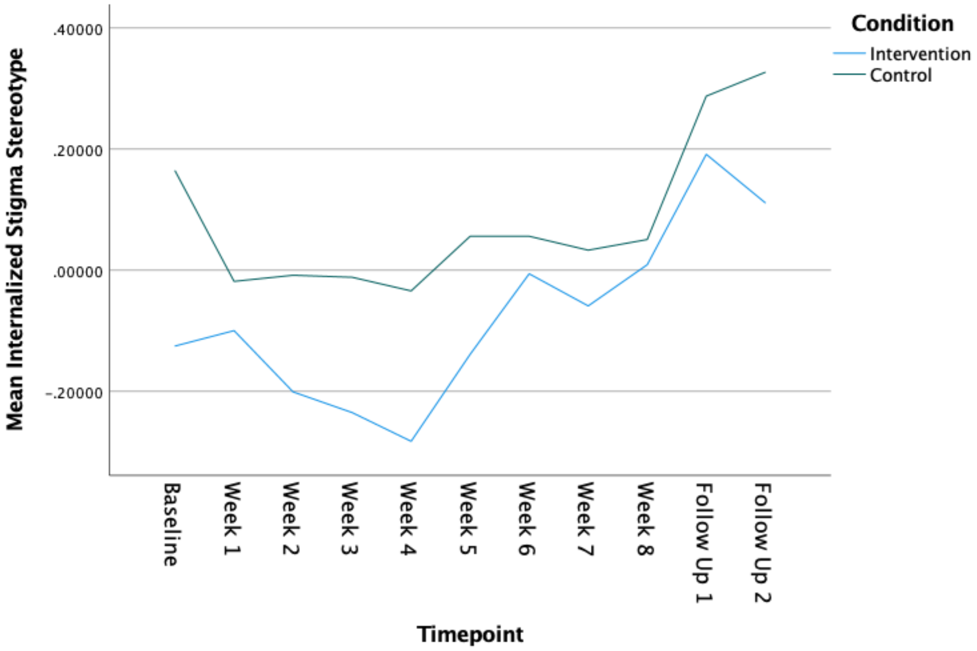

Social Connectedness

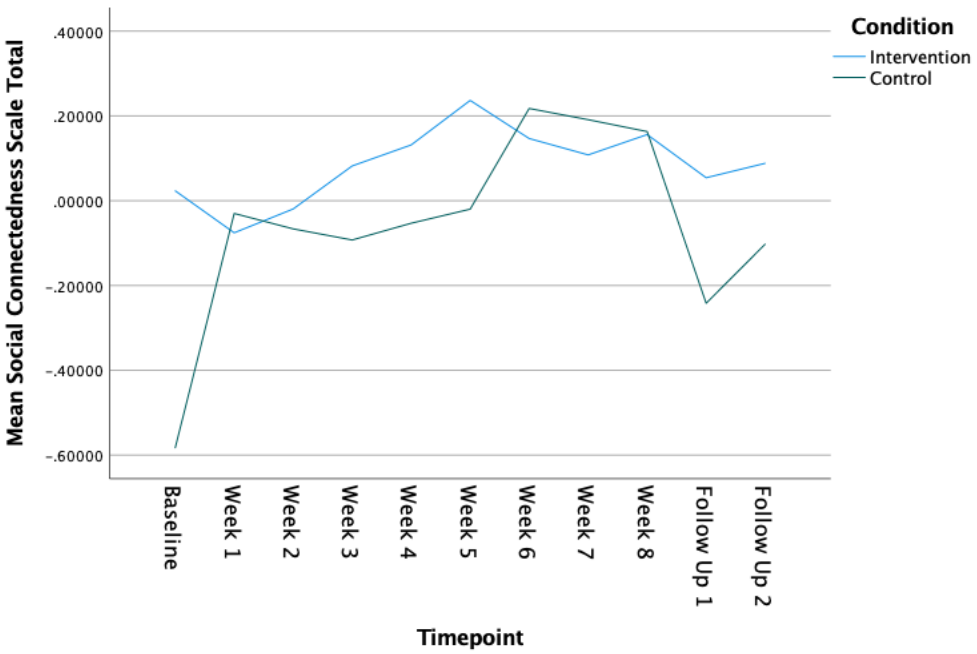
Mean Social Connectedness Scale Total
.40000
.20000
.00000
-.20000
-.40000
-.60000
Condition
Intervention
Control
Baseline
Week 1
Week 2
Week 3
Week 4
Week 5
Week 6
Week 7
Week 8
Follow Up 1
Follow Up 2
Timepoint
